# Personality predicts dispersal and settlement in a novel habitat—a mesocosm experiment

**DOI:** 10.1093/beheco/arag076

**Published:** 2026-07-09

**Authors:** J Gismann, T G G Groothuis, F J Weissing, M Nicolaus

**Affiliations:** Groningen Institute for Evolutionary Life Sciences, University of Groningen, Nijenborgh 7, 9747 AG, Groningen, The Netherlands; Department of Ecology and Complexity, Centre for Advances Studies Blanes (CEAB-CSIC), Carrer Accés Cala Sant Francesc, 14, 17300 Blanes, Spain; Groningen Institute for Evolutionary Life Sciences, University of Groningen, Nijenborgh 7, 9747 AG, Groningen, The Netherlands; Groningen Institute for Evolutionary Life Sciences, University of Groningen, Nijenborgh 7, 9747 AG, Groningen, The Netherlands; Groningen Institute for Evolutionary Life Sciences, University of Groningen, Nijenborgh 7, 9747 AG, Groningen, The Netherlands

**Keywords:** individuality, 3-spined stickleback, RFID, behavioral syndromes, colonization events

## Abstract

Individual phenotypes strongly affect dispersal. Yet studying the relationship between dispersal tendencies and personality under ecologically relevant conditions is challenging, especially in fish. Laboratory studies lack environmental complexity and scale, while field-based approaches are often unfeasible. We here mimicked dispersal events in fish; encompassing all 3 phases of dispersal (departure, transience, settlement) in a large experimental mesocosm containing breeding sites. Using 3-spined sticklebacks (*Gasterosteus aculeatus*) tagged with ‘Passive Integrated Transponders’ (PIT tags), we remotely monitored proxies of dispersal of 2 cohorts, each consisting of 40 individuals of known activity and aggressiveness scores, continuously over 2 wk. We tested whether personality variation explained differences in departure timing, transience movements, settlement success and spatial distribution along the environmental gradient. We found that more active/aggressive individuals dispersed quicker in the mesocosm, spent more time on territories and collected more eggs. However, we did not detect strong phenotype-environment correlations. Our findings emphasize the potential role of personality-dependent dispersal in population dynamics, with specific behavioral types driving dispersal and settlement success. Our mesocosm setup provides unique insights into the dispersal dynamics in a system where detailed observations across all dispersal stages are rare and difficult to obtain. Despite limitations, behavioral experiments in mesocosms serve as a valuable bridge between laboratory and natural systems.

## Teaser

Using a semi-natural experimental setup, we show that more active and aggressive sticklebacks dispersed sooner and explored faster. More active males were more successful at claiming territories and receiving eggs from females. These results highlight that personality traits shape dispersal behaviour and provide detailed insights into the role of behaviour in fish dispersal, a process typically difficult to study in such detail.

## Introduction

Individual phenotypes play a crucial role during dispersal events, when individuals move to, and ultimately settle in novel habitats. While many studies on dispersal focused on morphological or physiological traits such as body size or wing length in birds (Bowler and Benton 2005; Fargallo and López-Rull 2022), animals primarily interact with their environment through behavior (Wong and Candolin 2015). Accordingly, consistent individual differences in behaviors, which are often organized into suites of correlated behaviors (ie “animal personality”), are of major importance during dispersal events (Stamps and Groothuis 2010; Cote et al. 2011; Wolf and Weissing 2012; Mittelbach et al. 2014). Such “personality-dependent dispersal”, a specific case of a “dispersal syndrome”, is a common phenomenon that has repeatedly been observed across many taxa, including birds, mammals, fish, amphibians, and reptiles (Clobert et al. 2009; Cote et al. 2011; Cooper et al. 2017). For example, more active individuals, who are often more exploratory, bold, aggressive, or less social, typically disperse faster and farther. (Fraser et al. 2001; Cote and Clobert 2007; Cote et al. 2010; Chapple et al. 2012; Mullon et al., 2018). Dispersal syndromes are often assumed to be adaptive by functionally integrating traits that alleviate the costs of dispersal and/or increase the chances of establishment in novel habitats.

Dispersal can be divided into 3 consecutive phases: (i) departure, when individuals leave their place of birth (natal dispersal) or reproduction (breeding dispersal); (ii) transience, when individuals move to and explore novel environments (sometimes referred to as “floating”); and (iii) settlement, when individuals stop moving and settle for, eg reproduction (Clobert et al. 2004, 2009; del Mar Delgado and Penteriani 2008). Knowledge of the role that individual behavioral differences play in each of these dispersal phases is crucial to better understand the consequences of dispersal events in natural populations. For example, studies in western bluebirds (*Sialia mexicana*) showed that new habitats on the edge of the population's range are predominantly colonized by highly dispersive and aggressive males, which outcompete already established mountain bluebirds (*Sialia currucoides*) (Duckworth and Badyaev 2007; Duckworth et al. 2015). Once established, the proportion of aggressive types in the population quickly decreased due to negative density-dependent performance (Duckworth 2008). This example illustrates that initial settlement success in novel habitats is nonrandom with respect to behavior, leading to a spatial clustering of behavioral phenotypes and that dispersal syndromes play a major role in population dynamics.

Studying personality-dependent dispersal requires quantifying behavioral differences between dispersing and nondispersing individuals and accurately following the movement of animals over extended periods. Through the use of ringing and recent advances in miniaturized GPS tracking technology, researchers can decipher animal movement patterns on global scales in eg birds, large terrestrial mammals, or sea turtles (Fancy et al. 1988; Coyne and Godley 2005). Yet, for many smaller species, and especially in the underwater environment, comparable studies in the wild are more challenging. As a result, empirical studies explicitly linking personality traits to dispersal in fish are rare, but have shown that behavioral traits such as boldness and sociability can affect dispersal (Cote et al. 2010; Chapman et al. 2011). One alternative approach is to sample fish from newly-established populations (ie dispersers) and their source populations and to compare behaviors assessed in the lab (eg Lukas et al. 2021). While this approach may uncover population-level behavioral differences, it remains unclear whether and how preexisting behavioral differences directly contributed to dispersal. Further, because fish behavior is most frequently studied in the laboratory, translating results to natural conditions is often challenging. Semi-natural mesocosms, of greater complexity and scale than laboratory setups, that allow for detailed behavioral observations, can bridge the gap between laboratory experiments and field studies. Using such a mesocosm, we remotely tracked individual movements of large groups of small fish over several weeks. The mesocosm further allowed us to address a central and previously underexplored aspect of dispersal in fish: individuals' settlement success. Our study therefore spans all 3 phases of dispersal, capturing not only movement, but also settlement and breeding, thus directly assessing functional outcomes of personality-dependent dispersal.

In the mesocosm system (6 above-ground interconnected semi-natural ponds; for further details see Ramesh et al. 2023), we investigated how consistent differences in individual behaviors relate to proxies of dispersal tendencies in 3-spined sticklebacks (*Gasterosteus aculeatus*). To that end, we released mixed-sex groups of sticklebacks of known personality (activity and aggression assessed in the lab) into a novel environment and tracked individuals continuously using RFID (Radio-frequency Identification) technology across all 3 phases of dispersal. We focused on activity and aggression as these traits have repeatedly been found to differ between dispersers and nondispersers (O’Riain et al. 1996; Pocock et al. 2005). We asked whether and to what extent preexisting individual differences in activity and/or aggression contribute to dispersal-related decisions. Specifically, we asked, whether more active/aggressive fish initiate dispersal in the mesocosm quicker during the departure phase; how behavioral differences affect the way individuals sample the environment during transience, and, for males, which individuals are more successful in establishing a territory under competition in the settlement phase. We also explored whether behavioral tendencies predicted the distribution of males over different types of breeding territories, exploring the potential for phenotype-environment correlations (Edelaar and Bolnick 2012; Jacob et al. 2015).

## Materials and methods

### Experimental animals

We used 3-spined sticklebacks, which were born in semi-natural ponds (mesocosms) in summer 2020. These were obtained from natural mating of wild anadromous sticklebacks caught during inland migration in the northern Netherlands in spring 2020 (Nieuwe Statenzijl: (53°13′54.49″, 7°12′30.99″) (Ramesh et al. 2022). The fish were previously used in an experiment investigating the effects that predation cues (olfactory and physical) received during development have on behavior as sub-adults in late 2020 (Gismann et al., unpublished). Since all fish experienced the same treatment during development (3 wk of predation cues), we are confident that the previous use did not affect the experimental outcomes of the present study. Moreover, this treatment reflects ecologically relevant conditions, as fish in the wild are commonly exposed to both predation cues and actual predation risk.

In the previous experiment, all fish larger than 40 mm in total length were tagged with 8 mm PIT (“passive integrated transponder”) tags, allowing individual identification (Trovan, Ltd, Santa Barbara, California) by means of RFID (Radio-frequency Identification) technology (for tagging method see Ramesh et al. 2021). Between the preceding experiment and the present experiment, fish were housed in groups of 50 to 60 individuals in 4 large plastic semi-natural ponds (1.6 m diameter) outdoors, filled with 80 cm water from a nearby waterbody. In the present experiment, we re-used a total of 80 fish (20 from each housing pond), divided into 2 independent cohorts of 40 individuals each (20 females and 20 males per cohort). Test fish were housed according to pond of origin (*n* = 20) in aerated plastic buckets containing artificial plants and structures for shelter (40 cm diameter; filled with 25 cm of water from the mesocosm, refreshed every 2 to 3 d) placed next to the mesocosm. Fish were fed defrosted *Chironomus* larvae ad libitum daily. One male died over the course of the experiment in cohort 2, and was subsequently excluded from all analysis (total *n* = 79 fish).

### Ethical note

Housing of experimental animals, testing of behaviors and PIT tagging were in adherence with the project permit from the Central Committee on Animal Experiments (CCD, the Netherlands; license number: AVD1050020174084). We followed the 3R principles of animal experimentation by reusing PIT tagged individuals from a previous study. There, we did not detect any noticeable effects of PIT tagging and mortality from tagging was very low (< 1% within the first week of tagging, where risk of infection is highest [2/224 tagged fish died]). All procedures adhere to ASAB/ABS “Guidelines for the Ethical Treatment of Nonhuman Animals in Behavioral Research and Teaching” (https://doi.org/10.1016/S0003-3472(24)00376-2).

### Experimental overview

The experiment was conducted between April and June 2021 using 2 independent cohorts of sticklebacks (40 individuals each; 20 females and 20 males) tested consecutively in the same mesocosm setup. We continuously tracked individuals' movement using RFID technology throughout in the mesocosm environment. The setups included breeding sites monitored with RFID antennas that varied along a quality gradient, allowing assessment of male breeding site choice and settlement patterns. In cohort 2, we additionally monitored male mating success by counting the number of eggs collected in males' nests.

All individuals were tested twice for activity and aggression in the lab. For logistical reasons, the order of experimental procedures differed between cohorts: in cohort 1, lab-based behavioral assays were conducted before the mesocosm experiment (10 d prior), whereas in cohort 2, the mesocosm phase preceded the lab assays (lab assays started the day after the mesocosm phase).

### Laboratory tests activity and aggression

Fish were transported to the lab and placed in individual housing tanks (15 × 20 cm) containing a plastic plant for shelter and without visual access to neighbors for ∼24 h prior to behavioral testing. As fish were previously housed outdoors, they were kept under a light schedule following the natural sunlight hours in the Netherlands in May (∼15.5 h daylight; 06:00 to 21:30). Housing tanks were filled with a mixture of tap water and water from the mesocosm to a level of 10 cm. Fish was fed defrosted *Chironomus* larvae ad libitum daily after behavioral testing.

For the behavioral trials, fish were carefully transported to the test tanks in a water-filled beaker. Four test tanks (same dimensions as housing tanks but not containing any plastic plants and lined on all sides and bottom with opaque white plastic sheets and filled to a level of 8 cm) were placed together in a wooden testing box (60 × 80 × 80 cm) to limit outside disturbances during the trials, allowing 4 fish to be tested simultaneously. Testing boxes were illuminated with LED lights and trials were filmed from above using Raspberry Pi single-board computers fitted with a camera (Raspberry Pi NoIR Camera Board V2—8MP, Raspberry Pi Foundation, UK). Two testing boxes were used simultaneously, such that 8 fish could be tested at a time. After a 5-min acclimation time in the test tanks, the behavioral trials began.

“Activity” was quantified by the total distance moved (cm) in the empty test tank from 5-min video recordings tracked using the software “EthovisionXT” (Noldus Information Technology BV, Wageningen, The Netherlands). Directly thereafter, we remotely lifted an opaque plastic sheet to reveal a mirror on one of the short sides of the arena. The mirrors were 15 × 15 cm, thus covering the whole short side of the tanks. We assessed “aggression” by manually scoring antagonistic behaviors from 5-min video recordings after revealing the mirror as the number of bites and the time spent swimming head-on perpendicular to the mirror.

Fish sometimes interacted with the mirror in nonaggressive ways, such as slow parallel swimming or staying still next to the mirror image. As these behaviors are not consistent with antagonistic behaviors in stickleback, these were not scored as “aggression”. To avoid observer bias, all videos were scored by the same observer. Every fish was tested twice for both activity and aggression, with 1 d in between. For each of the 2 cohorts, we tested half of the fish on the same day, such that the laboratory tests took 4 d per cohort. Between trials, fish were housed in the same individual holding tanks as before the tests. Fish were returned to the same outdoor group housing after the laboratory experiments were completed.

Note on using mirror tests for measuring aggression: Despite their long tradition and frequency of application in a wide variety of fishes including sticklebacks (Tinbergen 1951; Moretz et al. 2007; Riebli et al. 2011; Josi and Frommen 2021; Axling et al. 2023), mirror tests are not without drawbacks and we briefly want to address some of these here. Firstly, mirror tests have the underlying assumption that individuals do not recognize themselves in their mirror image. While this assumption has recently been questioned for the cleaner wrasse *Labroides dimidiatus* (Kohda et al. 2023), there is currently no conclusive evidence for self-recognition in other fishes (although the question has not been addressed broadly, and some have argued that there is potential for false negatives; (Kohda et al. 2025)). Another main drawback lies in mirror-induced behavioral autocorrelation whereby more aggressive or more active individuals generate a more aggressive/active “opponent”. Although this drawback cannot be fully eliminated, we sought to reduce it by explicitly distinguishing aggressive displays from movement-based behaviors during the mirror test (ie we did not simply use time spent close to the mirror as a metric of aggression (described above), because this measure is strongly confounded by individual differences in general activity level). Consequently, we expect that any association between independently measured activity and aggression is less likely to reflect an artifact of the mirror setup and should more closely approximate a genuine relationship between these traits.

### The mesocosm system

**Figure 1:**
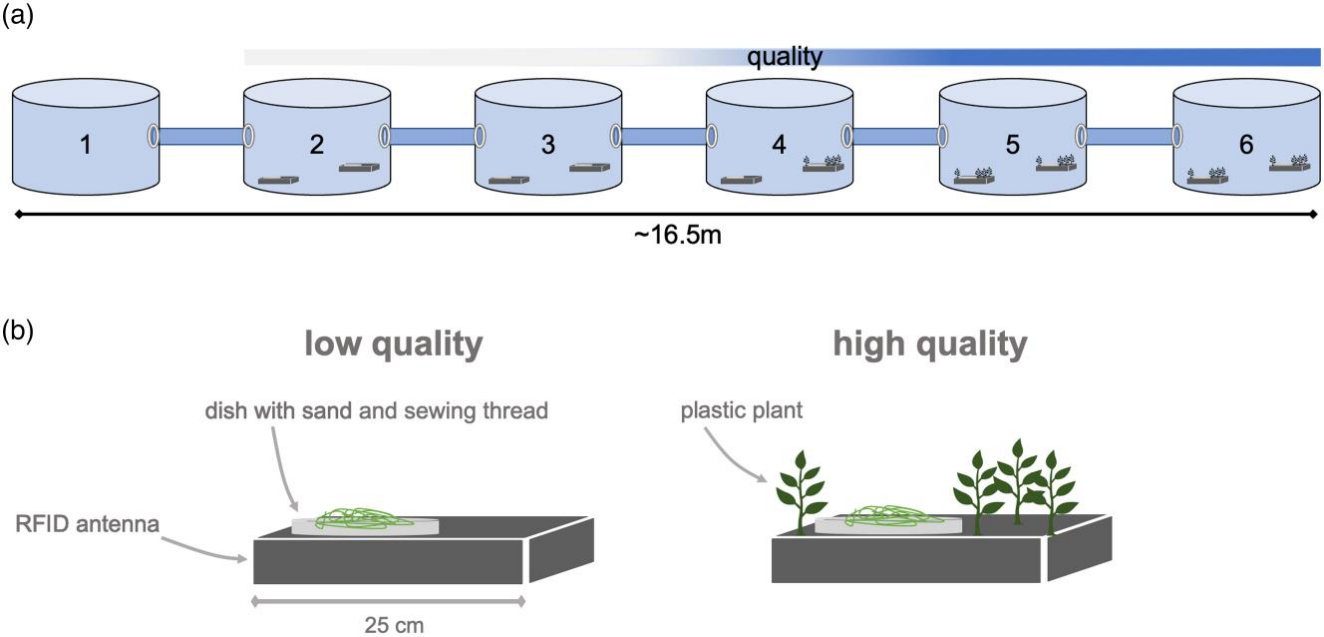
Mesocosm setup and breeding spots. a) Mesocosm setup containing 2 breeding spots in each pond (except pond 1), with a gradient from low- to high-quality breeding spots toward pond 6. Each pond additionally contained 3 small shelters (not depicted). b) Low- and high-quality breeding spots placed on top of ‘flatbed’ RFID antenna.

The mesocosm consisted of 6 linearly connected ponds of 1.6 m diameter filled to a height of approximately 60 cm with water from a nearby waterbody that is similar to natural conditions of wild sticklebacks in the Netherlands. The mesocosms are located outdoors under a tarp net to protect against avian predators. Temperature, weather (including rain) and light conditions vary according to the season. The mesocosms contained some naturally occurring aquatic invertebrates (eg insect larvae). No sediment was provided, to ensure that male sticklebacks could build nests only on the designated breeding spots. Ponds were connected via tubes (1.5 m length; 10 cm diameter) and fitted with RDIF antennas at the entrance and exit of each pond to automatically record the fish's movements between ponds (Fig. 1a). The RFID system was set to a sensitivity of 1 read/s. The reliability of the system was previously evaluated (Ramesh et al. 2023). All ponds contained 2 shelters in the form of artificial plants or plastic tubes.

Except for the starting pond, each pond contained 2 breeding spots. These breeding spots consisted of a plastic dish (18 cm diameter) filled with 2 cm of white aquarium sand (“lower-quality”) or sand with artificial plants (“higher-quality”). Male sticklebacks that build nests concealed in plants have previously been shown to exhibit higher mating success (Kraak et al. 2000). We thus expected that breeding spots containing plastic plants would experience higher competition. To aid nest building, all breeding spots in batch 2 additionally contained some sewing thread. All breeding spots were placed on top of “flatbed” RFID antennas that record the identity of fish that swam within 5 cm of the breeding spot (Fig. 1b). Hence, the presence of territorial as well as competing males could be identified. We created a gradient from lower- to higher-quality breeding spots the farther fish moved from the starting pond. Ponds 2 and 3 contained 2 lower-quality breeding spots, pond 4 contained 1 lower-quality and 1 higher-quality breeding spot, and ponds 5 and 6 two higher-quality breeding spots each (Fig. 1a).

Before testing, 40 fish (20 females, 20 males in each cohort) were released simultaneously into the first pond of the row, where they were given 4 d in cohort 1 and 7 d in cohort 2 to acclimatize. Next, the connection to the other ponds was opened, marking the start of the experiment (17:20 and 17:06 in cohort 1 and cohort 2, respectively), and movement and mating success was monitored for 17 d (10 May 2021–26 May 2021) and 14 d (01 June 2021–14 June 2021) in cohort 1 and 2, respectively.

To quantify dispersal behavior, we extracted multiple outcome parameters from the fish's RFID movement data in the mesocosm. For clarity, we refer to these measurements in terms of conceptual “dispersal phases” (departure, transience, settlement), which represent typical stages of dispersal rather than discrete experimental manipulations. One or more parameters were assigned to each phase to capture the corresponding aspect of behavior.

#### Departure phase—latency to exit the starting pond

From RFID reads we measured each fish's latency to exit the first pond as the time interval between the start of the experiment (ie opening the connector from pond 1 to the other ponds) and reaching pond 2. As all fish exited pond 1, we did not have to define a latency time for fish that never exited the first pond.

#### Transience phase—movements in the mesocosm

From the RFID reads we extracted several measures related to the fish's behavior during the transience phase. We extracted movement in the mesocosm as the number of crosses between ponds and crosses between breeding spots within the first 24 h after leaving the starting pond (on an individual basis, ie based on each individual's latency to exit the first pond). We chose 24 h as the cutoff for the transience phase for several reasons. Firstly, despite its relatively large spatial scale (∼16.5 m), the system can be explored rapidly (eg on average fish had about 60 pond crosses on the first full day of the experiment in cohort 1). Note that these crosses do not necessarily correspond to movement throughout the whole system, but can also occur due to eg movement back and forth between 2 adjacent ponds. Secondly, except for 16 individuals, all fish explored the full system (ie reached the last pond) within 24 h of exiting the first pond. Lastly, we observed that many males started to settle on territories already within the first day of the experiment. Furthermore, as a measure of overall exploration speed, we calculated the *latency to reach the last pond*, as the time it took each fish to reach pond 6 after starting the experiment. Overall, 5 fish never reached the last pond and were excluded from this aspect of the analysis.

#### Settlement—territory establishment (cohorts 1 and 2)

To assess whether and which males successfully established a territory, we applied a number of criteria, based on the reads collected from the antennas underneath the breeding spots. First, as a proxy for the time each male spent on each breeding spot, we counted each male's total number of daily reads on each breeding spot antenna, (starting from the second day of the experiment). The male with the most reads on a given antenna on a given day was deemed the “occupant” of that specific breeding spot on that day. While this technically allows for males with only very few reads to be assigned the occupant of a specific breeding spot, we found that males typically produced large numbers of reads per day. The mean number of reads by the male with the most reads on a given day across all breeding spots was 8,539.2 (SD = 8,892.8) and 13,009.7 (SD = 14,035.9) in cohort 1 and cohort 2, respectively. Male sticklebacks need some time to establish a territory and to build a nest (Mori 1993). In a laboratory study without nesting competition, sticklebacks took for example between 1 and 4 d for nest building, with a median of 1 d (Bakker and Mundwiler 2021). We thus considered males as having successfully established a territory if they were the occupant of a breeding spot on 3 or more consecutive days. We subsequently counted the number of days that each male spent on a territory (only for each male's first instance of establishing a territory, as we deemed it the more important event regarding male-male competition).

We defined a breeding spot as contested when on the same day 2 (or more) males had a comparable number of PIT tag reads on that breeding spot. Specifically, we defined a breeding spot as “contested” on a given day if the male with the second-most reads had at least half as many reads as the breeding spot occupant (most read male). In Figure S2, such contested days on a breeding spot are marked by a red cross, as they provide useful information on conflicts over breeding spots.

#### Settlement—mating success (cohort 2)

In cohort 2, we monitored all breeding spots closely for the presence of eggs, knowing that male sticklebacks can collect several clutches from different females (Mori 1993; Kraak et al. 1999; Ishikawa and Mori 2000). We assessed each breeding spot every 2 to 3 d, by briefly lifting them to the water surface and carefully checking for eggs in the nests. When nests were empty, we placed the breeding spot without any further disturbance back on the RFID antenna in the pond. When present, eggs were removed from the nest and counted, and the (partially destroyed) nest was returned to the pond. The eggs were assigned to the male that held the corresponding territory on the day of egg detection.

### Statistical analyses

#### Laboratory tests

To quantify consistent individual differences in activity and aggression, we calculated “raw” repeatabilities (without the inclusion of fixed effects) using the “rptR” package (Stoffel et al. 2017). For activity, we specified a generalized linear mixed model (GLMM) with a Gaussian error distribution and individual ID as a random effect. In the aggression models, we used a Gaussian error distribution for the time spent vigorously swimming and Poisson error distribution with a square root link function for the number of bites and included individual ID as a random effect. We calculated repeatabilities (R) and their 95% confidence intervals (CI) with 1,000 bootstraps. For the number of bites, we report “latent” (link-scale) approximations (Stoffel et al. 2017). We calculated Pearson correlation coefficients to quantify correlations between behaviors.

#### Personality-dependent dispersal and territory establishment

To test for an association between individual behavioral phenotype and dispersal-related traits, we ran separate linear models for each dispersal behavior proxy measured in the mesocosm. We used *mean activity* scores (individual mean over the 2 trials) as a proxy of personality to avoid collinearity issues (levels of activity and aggression are positively correlated; see Results). Thus, throughout this study, individuals classified as more active tended to also be more aggressive. Initially, all models included an interaction between *activity* and *sex* (sex fitted as a factor with *female* used as reference category). The interaction was nonsignificant, and models including the interaction term did not perform better according to Akaike information criterion (ΔAIC < 2). We therefore removed the interaction term and retained *sex* as a fixed effect in all final models (except for the male-only model on the days spent on a territory). All models contained the experimental *cohort* as a fixed effect (with cohort 1 used as reference category), to control for potential differences between the 2 batches of fish. For models on the latency to leave the first pond and the latency to reach the last pond (pond 6), Gaussian error distributions were fitted. Data were log-transformed in the model on the latency to reach the last pond. For the number of pond crosses (and breeding spot crosses) during transience and for the number of days on a territory, a negative binomial family was fitted to handle overdispersion, using the glmmTMB package (Brooks et al. 2017) (Table 1). Welch's 2-sample *t*-tests and Wilcoxon rank-sum tests were performed to test if mean activity level differed between males that acquired a territory or not, acquire eggs or not, and obtained a low quality or a high-quality territory. Preliminary analysis revealed that size did not correlate to any lab behaviors (activity and aggression) or dispersal-related traits (latency to leave the first/reach the last pond), and we thus did not include size as a fixed effect in the linear models. Ideally, we would have investigated behavioral syndromes using multivariate mixed-effects (GLMM) models to estimate trait covariances while avoiding error propagation (“stats-on-stats”). However, multivariate models are data hungry and require repeated measurements within individuals to decompose variance components (Dingemanse and Dochtermann 2013). Given our data structure (1 dispersal proxy per fish) and the relatively limited sample size (79 fish), GLMMs could not be used here. We thus openly state these potential limitations as advocated by Dingemanse and Wright (2020).

**Table 1:** Model summary of generalized linear models of latency to leave the first pond in hours, hours to reach pond 6 (log-transformed), number of pond crosses during the transience phase, and number of days spent on a territory.

	Departure latency		Latency to pond 6		Transience pond crosses		Days on territory	
Predictors	Estimates	CI	Estimates	CI	Incidence Rate Ratios	CI	Incidence Rate Ratios	CI
Intercept	61.330***	31.583–91.076	4.152***	3.445–4.858	147.453***	90.484–240.288	0.862	0.242–3.070
Activity	−0.037*	−0.073 – −0.001	−0.001*	−0.002 – −0.000	1.000	0.999–1.000	1.002**	1.001–1.003
Sex (M)	−11.264	−29.623–7.095	−0.111	−0.541–0.320	0.723	0.510–1.025	…	…
Cohort (2)	12.477	−5.726–30.680	0.142	−0.284–0.569	0.645*	0.454–0.917	0.611	0.237–1.574
Observations	79		74		79		39	

^*^ *P* < 0.05 **P < 0.01 ***P < 0.001.

Mean activity scores, sex of the fish and experimental cohort were included as fixed effects with sex (F) and cohort (1) being used reference categories. Estimates or Incidence Rate Ratios (for neg. binomial model families) are given with their 95% CI.

## Results

### Laboratory tests

**Figure 2:**
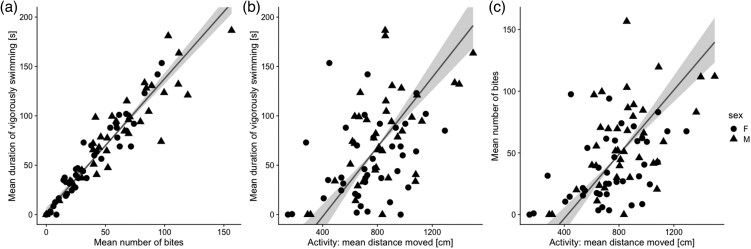
Correlations between behaviors measured in the laboratory. a) Mean time vigorously swimming plotted against the mean number of bites. b) Mean time spent vigorously swimming plotted against mean activity (distance moved in cm). c) Mean number of bites plotted against mean activity. Standardized major axis (SMA) regression fits are displayed with 95 % confidence intervals (grey shaded area). Females are represented by circles, males by triangles. Sample size for all plots is *n* = 79.

Repeatability between trial 1 and trial 2 was moderate across behaviors: activity (R [95% CI] = 0.511 [0.335, 0.67]), number of bites (R [95% CI] = 0.393 [0.185, 0.546]), and time spent vigorously swimming (R [95% CI] = 0.321 [0.104, 0.503]). CI overlapped, indicating no detectable differences among traits. For subsequent analysis, we calculated individual means in activity, number of bites, and time spent vigorously swimming over the 2 trials. Both measures of aggression were highly correlated (Pearson *ρ* = 0.94, *P* < 0.001; Fig. 2a). Mean activity was positively correlated with both measures of aggression (mean activity—mean time spent vigorously swimming: Pearson *ρ* = 0.47, *P* < 0.001 [Fig. 2b]; mean activity—mean bites: Pearson *ρ* = 0.50, *P* < 0.001 [Fig. 2c]), indicating the existence of an activity-aggression syndrome. Visual examination of the relationship between mean activity and mean aggressive behaviors suggests, that the observed correlations result in part from individuals at both extremes of the activity/aggressiveness distributions. The most active individuals were consistently highly aggressive, and vice versa. Males were overrepresented among the fish with the highest mean aggression and generally males were more aggressive than females (mean bites males = 80.3; mean bites females = 53.2), while females were less active and overrepresented among the fish with the lowest mean activity scores (mean activity males = 839.9; mean activity females = 739.9).

### Departure

**Figure 3:**
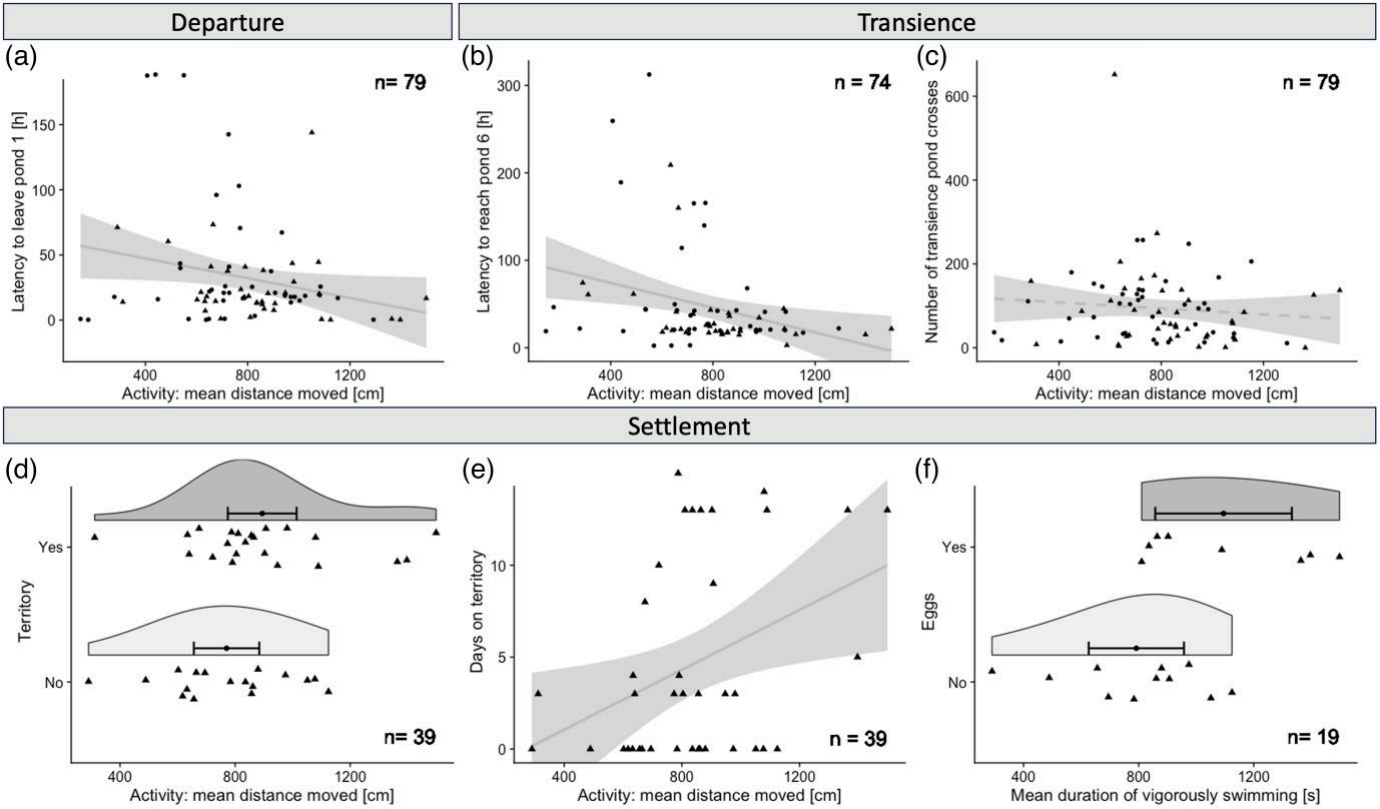
Relationship between activity level and behavior in the departure, transience, and settlement phase. Departure phase: a) Latency to leave the first pond plotted against mean activity. Transience phase: b) Latency to reach the last pond plotted against mean activity. c) The number of pond crosses during transience plotted against mean activity. Settlement phase: d) Distribution of mean activity scores of males that successfully acquired a territory (‘Yes’) and males that did not (‘No’). e) The number of days on a territory plotted against mean activity. f) Distribution of mean activity scores of males that acquired eggs (‘Yes’) and males that did not acquire eggs (‘No’) in cohort 2. Solid lines represent linear regression lines with 95% confidence intervals and dashed lines indicate that the regression coefficient did not differ significantly from zero. In d) and f), data points with density kernels are plotted with mean and 95% confidence intervals. Females are represented as circles and males as triangles.

There was considerable variation in the departure time from the first pond, yet all fish in the experiment eventually left the first pond. The fastest fish left within 15 min (0.26 h), while the slowest took almost 8 d (188.5 h) (median = 18.4 h; mean = 32.6 h). More active individuals exited the first pond more quickly than less active ones (Fig. 3a). The model explained a modest proportion of variance in departure latency (*R*^2^ = 0.095), and the effect was independent of sex and experimental cohort (Table 1). It is noteworthy, that not all fish fitted to this general pattern. For example, the 2 fish that displayed the lowest activity levels in the lab, were among the first to leave the first pond. The observed pattern seems may in part be driven by fish with high activity scores, which consistently had a low latency to leave the first pond (ie variation in latency was greater among less active than among more active individuals). Furthermore, fish that took to the longest to depart were predominantly female.

### Transience

All but 5 fish entered the last pond (Pond 6). The latency to reach the last pond ranged from 2.4 h to 312.5 h (∼13 d) (median = 22.4 h; mean = 47.6 h). We found that more active fish were faster in reaching the last pond (Fig. 3b; Table 1). The model explained a modest proportion of the variation in latency to reach the last pond (*R*^2^ = 0.082). The latency to reach the last pond followed a similar distribution as the latency to leave the first pond. Again, variation in latency was greater in less active fish, while the fish with high activity scores were consistently fast to reach the last pond. The fish with the most extreme latency values were mostly female (7 out of 9 fish with latency > 5,000 min). The number of crosses between ponds (Fig. 3c) and the number of breeding spot crosses (Figure S1a) during the transience phase (the first 24 h after departure from the first pond) did not covary with mean activity. The model of pond crosses during the transient phase explained little variation (McFadden's pseudo-*R*^2^ = 0.01), but indicated that fish in cohort 2 moved less between ponds than fish in cohort 1 (Figure S1b; Table 1). No effect of sex was observed in either of the aforementioned models (Table 1).

### Settlement

Males that successfully acquired a territory tended to show higher levels of activity (mean = 894.0) than nonsuccessful males (mean = 770.1), but this difference was not statistically supported (Welch *t*-test: *t*(36.84) = −1.58, *P* = 0.12; Wilcoxon rank sum test: *W* = 137, *P* = 0.16). Yet, considering all males, more active fish spent more days on territories than less active fish (Fig. 3e; Table 1), although the model explained only a small proportion of variation in days on territory (McFadens pseudo-*R*^2^ = 0.033). In cohort 2, males that acquired eggs tended to be more active than males that did not (Fig. 3f). The mean activity of males that acquired eggs was 1,095.1 (SD = 284.0), vs. 792.0 (SD = 246.6) for those that did not. This difference was statistically significant using a Welch *t*-test (*t*(13.86) = −2.43, *P* = 0.03) but not with a Wilcoxon rank-sum test (*W* = 22, *P* = 0.08). We report both results to provide a transparent view of the data and to indicate that the trend is robust across parametric and nonparametric analyses. Figure S2 provides a more detailed picture of the dynamics of territory establishment in our experiment.

### Territory quality and spatial distribution of behavioral phenotypes

Over both cohorts we found 12 instances of territories established on higher-quality breeding spots and 16 instances of territory establishment on lower-quality breeding spots. On average, males spent more time on higher- than on lower-quality breeding spots (mean time on higher-quality breeding spots: 9.83 d [total of 118 cumulated days]; and mean time on lower-quality breeding spots: 5.75 d [total of 92 cumulated days]) (*t*-test: *t*(26) = 2.58, *P* = 0.02; Wilcoxon rank sum test: *W* = 139.5, *P* = 0.04), explaining the higher total number of established territories on lower-quality breeding spots.

Breeding spots were arranged such that fish were presented with a gradient from low- to high-quality. However, we did not detect any phenotype-environment correlation (Figure S3). Males on high- and low-quality breeding spots did not differ in mean activity (mean activity on high-quality = 863.3 vs. mean activity on low-quality = 996.2; *t*-test: *t*(26) = −1.18, *P* = 0.25; Wilcoxon rank sum test: *W* = 78.5, *P* = 0.43) but differed however in mean aggression: Males on high-quality breeding spots were less aggressive than males on low-quality breeding spots (mean time spent vigorously swimming on high-quality = 73.3 s, vs. low-quality = 111.4 s; Welch *t*-test: *t*(20.99) = −2.38, *P* = 0.03; Wilcoxon rank sum test: *W* = 47.5, *P* = 0.03; Figure S4).

## Discussion

This study aimed to investigate how individual differences in activity and aggression contribute to dispersal-related choices and outcomes over the different phases of dispersal in 3-spined sticklebacks. As previously reported in this species (Bell and Stamps 2004; Dingemanse et al. 2007), our study confirms that more active fish were more aggressive. Furthermore, confirming our hypothesis, we found evidence for personality-based dispersal in all 3 phases of dispersal. Compared with less active-aggressive individuals, more active-aggressive fish departed quicker from the starting pond and moved faster during the transience phase, although behavioral types did not differ in the way they sampled the environment. In males, personality did not predict territory acquisition but, among successful settlers, more active-aggressive males overall spent more time on a territory, and, in the second cohort, acquired more eggs. Territory quality weakly affected the spatial distribution of aggressive phenotypes, thereby not providing strong support for the existence of phenotype-environment correlations along the manipulated gradient. We discuss below the implications of our findings.

We show that high levels of activity (and aggression) were positively associated with proxies of faster dispersal and mating success. Uncertainty around the mechanisms underlying such behavioral correlations often limits our understanding of the eco-evolutionary implications of dispersal syndromes (Nicolaus et al. 2022). For example, behavioral differences between dispersers and residents might rather be the consequence than the cause of dispersal, if dispersers adjust their behavior to conditions experienced during or after dispersal (Holekamp 1986; Hoset et al. 2011). Here we tested aggression and activity shortly before the dispersal experiment in cohort 1, and shortly after the dispersal experiment in cohort 2. Because the same relationships were found between activity/aggression and a proxy of dispersal across both experimental cohorts, it is unlikely that the personality-dispersal syndrome results from short-term plasticity. Instead, our study highlights that in our population, preexisting personality variation led to a subset of the population driving dispersal. Our results thus indicate that, in the wild, newly established stickleback populations may differ in the composition of behavioral phenotypes compared with their source population. Since dispersal related traits often have a genetic basis (Saastamoinen et al. 2018), personality-dependent dispersal can result in founder effects with important eco-evolutionary consequences for the populations (Via 1999; Santos et al. 2012).

Stickleback dispersal in the wild likely occurs over much larger distances than measured in our mesocosm system. Therefore, we recognize that our measures of dispersal should be viewed as proxies. It is also important to distinguish between routine daily movements or activity, and true dispersal. In a mark-transplant-recapture study conducted on wild sticklebacks, Bolnick et al. (2009) recaptured fish up to 400 m from their release point after just 4 d, indicating that sticklebacks can cover significant distances within a single day. In our experiments, a small proportion of fish did not leave the starting pond within the first day, and some took several days to do so. This suggests that individual variation in the latency to leave the initial pond likely reflects differences in dispersal propensity rather than daily activity. Ideally, a setup would impose higher costs to dispersal, such that only a subset of individuals disperses at all, to better distinguish variation in speed of dispersal from variation in propensity to disperse, which is expected to have larger eco-evolutionary consequences.

We further found that more active and more aggressive phenotypes were not only more dispersive but also more likely to collect eggs during reproduction under competition. Higher levels of aggression can be beneficial under intraspecific competition and can result in higher quality or more territories for male sticklebacks (Whoriskey and FitzGerald 1994). As a result, the proportion of more active-aggressive individuals could increase in the next generations of the newly established population, providing that dispersal-related traits have a genetic basis (Saastamoinen et al. 2018) and that sticklebacks mate assortative based on personality (but females have also been shown to avoid very aggressive males (Ward and FitzGerald 1987)). Invasive cane toads (*Rhinella marina*) in Australia provide an example of such a phenomenon where individuals at the front of the colonization were more exploratory and risk-prone compared with those from core-populations (Gruber et al. 2017). Differential selection likely favored dispersal-related traits that aid in the search for food and shelter. The idea that the frequency of exploratory/risk-prone phenotypes may increase over time, thereby accelerating the evolution of dispersal-enhancing traits, warrants future testing (Cayuela et al. 2020).

One intriguing question arising from our results is thus why not all individuals in the population display these beneficial dispersal-related behaviors. Several mechanisms can explain the persistence of behavioral heterogeneity within newly established populations. Firstly, dispersal is often costly (Bonte et al. 2012). Predation pressure, for example, may be stronger on more dispersive individuals (Wolf and Weissing 2012). Secondly, selection on dispersal syndromes likely fluctuates and/or is context-dependent. For example, in pied flycatchers (*Ficedula hypoleuca*), the strength of the association between dispersal and aggression and its associated fitness differed between years, which highlights that specific behavioral phenotypes are favored in some years but not in others (Nicolaus et al. 2022). Similarly, in western bluebirds the fitness of highly aggressive dispersing individuals was shown to be density-dependent (Duckworth 2008). Additionally, the structure of behavioral syndromes can be less stable than typically assumed. A long-term study on collared flycatchers (*Ficedula albicollis*) demonstrated that the strength and direction of behavioral correlations generally differed between the years (Garamszegi et al. 2015). Accordingly, the most active-aggressive-dispersive individuals in our experiment may thus only be favored during the initial phase of colonization/settlement and will likely be subjected to heterogeneous selection over time. Other behavioral types may be favored at a later point in time when, for example, population densities change.

Our results did not reveal personality-related differences in sampling of the environment during the transience phase. Following theories on coping styles, 1 could have expected that highly active, aggressive and dispersive individuals (“proactive”), would explore their environment quickly but superficially, whereas “reactive” individuals would explore slowly and thoroughly (Koolhaas et al. 1999; Réale et al. 2010). The lack of personality-related differences in sampling could be an artifact of the timing of the experiment, which took place relatively late in the sticklebacks' reproductive period. The fact that all but 1 breeding spot were occupied from the first day of the experiment in cohort 2 highlights that males attempted to reproduce as soon as the opportunity arose, without prolonged sampling. The motivation to reproduce may have masked individual variation in exploration. Additionally, mesocosm complexity may have been insufficient to demand thorough exploration.

Against our expectation, we did not detect a clear phenotype-environment correlation along the environmental gradient: male aggression but not activity level differed between high- or low-quality breeding spots and, while all breeding sites were ultimately occupied, males spent more time on high-quality ones. Thus, opposed to previous studies (Pearish et al. 2013; Bensky and Bell 2022), we can here not conclusively discern whether different personality types exhibited preferences (or outcompeted others) for specific habitats. In 3-spine sticklebacks, it was shown that personality types consistently used different microhabitats in the wild based on diet (Pearish et al. 2013). Such phenotype-environment correlations can lead to feedback loops which affect levels of phenotypic variation within a population and can be an important source of variation in fitness (Wolf and Weissing 2010). Additionally, if individuals are able to select habitats that match their phenotype (“matching-habitat choice”; Edelaar et al. 2008) selection will be relatively weak compared with individuals that are excluded from their preferred habitats due to eg social competition. The absence of strong phenotype-environment correlations in our experiment could thus lie in our choice of the environmental gradient. It is plausible that the pressure for timely reproduction and competition for breeding opportunities were so large, that males simply did not discriminate between breeding sites.

Altogether, our findings add to the growing literature on dispersal syndromes, showing how personality traits might be linked to dispersal tendencies and associated with differential fitness outcomes. We present evidence of such a syndrome in a small fish where individual-level observations in the wild are limited. Mesocosm experiments promise exciting avenues for future experiments, though they do not fully capture important ecological pressures that might counteract the advantages of fast dispersal, such as increased predation, energetic limitations, or heterospecific competition. More long-term work quantifying fluctuating selection pressures is thus warranted given population's large behavioral variation.

## Supplementary Material

arag076_Supplementary_Data

## Data Availability

Analyses reported in this article can be reproduced with the data provided by Gismann et al. (2026).
