# Supplementary material for: Personality predicts dispersal and settlement in a novel habitat—a mesocosm experiment
Source: Behav Ecol. 2026 Jul 9;37(5):arag076. doi: 10.1093/beheco/arag076 (PMC13448286; doi:10.1093/beheco/arag076)
Supplement: Supplementary file 1 [file arag076_supplementary_data.zip › Supplementary_ material_R3.docx]

# Supplementary material for `Personality predicts dispersal and settlement in a novel habitat – a mesocosm experiment`

**Fig. S1: Additional plots on transience behaviours.** **(a)** Number of crosses between breeding spots in the first 24 h after departure plotted against mean activity. We did not find that more active fish moved more often between breeding spots than less active fish. The dashed grey line (with 95% confidence interval) indicates that the regression coefficient did not significantly differ from zero. Females are represented as circles and males as triangles. **(b)** The number of pond crosses during the transience phase plotted by cohort, showed that fish in cohort 1 crossed more often than in cohort 2. Data points with density kernels are plotted with mean and 95% confidence intervals.

**Table S1:** Results of linear regression analyses for the dependence of the number of crosses between breeding spots during the transience phase on mean activity scores. We used generalised linear mixed models including the sex of the fish and the experimental cohort as fixed effects. Incidence Rate Ratio for neg. binomial model family are given with their 95% confidence intervals. Sex (F) and Cohort (1) are the reference categories.

**Figure S2. Overview of male territory establishment and breeding spot occupancy (a)** Territory establishment for each male that established a territory or had a contested day in cohort 1. **(b)** Succession of occupancy on each breeding spot in cohort 1. **(c)** Territory establishment for each male that established a territory or had a contested day in cohort 2. **(d)** Succession of occupancy on each breeding spot in cohort 2. Orange dots represent the starting day of territory occupancy and black dots represent the end day. Breeding spot names represent pond number, breeding spot number within pond (e.g., 2.1= pond 2 breeding spot 1) and quality (L= low quality; H=high quality). Red crosses represent contested days on breeding spots. Colours in (b) and (d) correspond to the colours of the males in (a) and (c), respectively.

For each of the 20 males in cohort 1 (Fig. S2a) and each of the 19 males in cohort 2 (Fig. S2c), this figure shows on which experimental day a male was classified as the “owner of a territory”. It also shows for both cohorts which of the ten breeding spots were “occupied” or “contested” on each experimental day, and which male was the occupant (Fig. S2b & S2d). In cohort 1 (Fig. S2a), 13 out of 20 males established a territory, while in cohort 2 (Fig. S2c), only 9 out of 19 males established a territory. Generally, in cohort 2, territory establishment happened more quickly and territories were held for a longer period than in cohort 1. In fact, 7 of the 10 breeding spots had the same occupant throughout the whole experiment in cohort 2 (Fig. S2d), while breeding spot occupation was much more dynamic in cohort 1 (Fig. S2c).

Some interesting observations can be distilled from the detailed account of breeding spot occupancy in Figure S2. For example, in cohort 1, *male 1R* first occupied breeding spot *4.1-L* for 4 days and on the third day (18.05.) also began to occupy breeding spot *4.2-H*, the higher-quality breeding spot in the same pond (Fig. S2a & S2b). On the following day, it left the lower-quality breeding spot and remained on the one of higher quality until the end of the experiment. The lower-quality breeding spot was subsequently occupied by *male 1K*. Similarly, in cohort 2, *male 2F* occupied both breeding spots in pond 2 on the first three days of the experiment (*2.1-L* and *2.2-L*; Fig. S2d). On the subsequent days of the experiment, the male seems to have ‘focused’ on breeding spot *2.1-L*, which it occupied until the end of the experiment. Interestingly, no other male managed to establish a territory on the other, now vacant, breeding spot within the same pond. The fact that breeding spot *2.2-L* was contested on four days in this ‘vacant’ period indicates that several males tried to settle there, but without success.

**Fig. S3: Relationship between male activity and territory quality.** L= lower-; H= higher-quality territories. Data points with density kernels are plotted with mean and 95% confidence intervals. Males on higher-quality breeding spots had a mean activity score of 863.3, and males on lower-quality breeding spots had a mean activity score of 996.2 (t-test: t(26) = -1.18, p = 0.25; Wilcoxon rank sum test: W = 78.5, p = 0.43). In total we found 28 instances of territory establishment.

**Fig. S4: Relationship between male aggression and territory quality.** L= lower-; H= higher-quality territories. Data points with density kernels are plotted with mean and 95% confidence intervals. We found that male son lower-quality territories were more aggressive than males on higher-quality territories. Males that occupied higher-quality breeding spots had a mean time spent vigorously swimming of 73.3s, while males that occupied lower-quality breeding spots had a mean time spent vigorously swimming of 111.4s) (Welch t-test: t(20.99) = -2.38, p = 0.03; Wilcoxon rank sum test: W = 47.5, p = 0.03). In total we found 28 instances of territory establishment.
